# Prediction of hub genes associated with intramuscular fat content in Nelore cattle

**DOI:** 10.1186/s12864-019-5904-x

**Published:** 2019-06-25

**Authors:** Danielly Beraldo dos Santos Silva, Larissa Fernanda Simielli Fonseca, Daniel Guariz Pinheiro, Maria Malane Magalhães Muniz, Ana Fabrícia Braga Magalhães, Fernando Baldi, Jesus Aparecido Ferro, Luis Artur Loyola Chardulo, Lucia Galvão de Albuquerque

**Affiliations:** 1 School of Agricultural and Veterinarian Sciences, São Paulo State University (UNESP), Jaboticabal, SP Brazil; 2 National Council for Scientific and Technological Development (CNPq), Brasilia, DF Brazil; 3 School of Veterinary and Animal Science, São Paulo State University (UNESP), Botucatu, SP Brazil

**Keywords:** Bovine, Co-expression, Genes, Lipids, Transcriptome

## Abstract

**Background:**

The aim of this study was to use transcriptome RNA-Seq data from *longissimus thoracis* muscle of uncastrated Nelore males to identify hub genes based on co-expression network obtained from differentially expressed genes (DEGs) associated with intramuscular fat content.

**Results:**

A total of 30 transcriptomics datasets (RNA-Seq) obtained from *longissimus thoracis* muscle were selected based on the phenotypic value of divergent intramuscular fat content: 15 with the highest intramuscular fat content (HIF) and 15 with the lowest intramuscular fat content (LIF). The transcriptomics datasets were aligned with a reference genome and 65 differentially expressed genes (DEGs) were identified, including 21 upregulated and 44 downregulated genes in HIF animals. The normalized count data from DEGs was then used for co-expression network construction. From the co-expression network, four modules were identified. The topological properties of the network were analyzed; those genes engaging in the most interactions (maximal clique centrality method) with other DEGs were predicted to be hub genes (*PDE4D, KLHL30* and *IL1RAP*), which consequently may play a role in cellular and/or systemic lipid biology in Nelore cattle. Top modules screened from the gene co-expression network were identify. The two candidate modules had clear associated biological pathways related to fat development, cell adhesion, and muscle differentiation, immune system, among others. The hub genes belonged in top modules and were downregulated in HIF animals. *PDE4D* and *IL1RAP* have known effects on lipid metabolism and the immune system through the regulation of cAMP signaling. Given that cAMP is known to play a role in lipid systems, *PDE4D* and *IL1RAP* downregulation may contribute to increased levels of intracellular cAMP and thus may have effects on IF content differences in Nelore cattle. *KLHL30* may have effects on muscle metabolism. Klhl protein families play a role in protein degradation. However, the downregulation of this gene and its role in lipid metabolism has not yet been clarified.

**Conclusions:**

The results reported in this study indicate candidate genes and molecular mechanisms involved in IF content difference in Nelore cattle.

**Electronic supplementary material:**

The online version of this article (10.1186/s12864-019-5904-x) contains supplementary material, which is available to authorized users.

## Background

Intramuscular fat (IF) content is an important meat quality trait that represents the amount of fat accumulated between muscle fibers or inside muscle cells; it is the sum of phospholipids and triglycerides. IF plays a key role in meat quality, as it is mainly responsible for the palatability, tenderness, and nutritional value of meat [1–3]. IF content may be measured via chemical methods (which evaluate total lipids) or by marbling scores (which may be assessed after slaughter or by ultrasonography). IF content is a polygenic trait regulated by many genes involved directly or indirectly in fat metabolism [4].

Next generation sequencing applied to tissue transcriptomes (RNA-Seq) is an approach for screening the expression of functional candidate genes and consequently is possible identify important molecular mechanisms that generate variation in pathways resulting in different tissue phenotypes [5, 6]. This technology has been used to identify differentially expressed genes (DEGs) in muscle tissue of Nelore cattle divergent to different meat traits, as such: tenderness [7], fatty acid composition [8], intramuscular fat content (measured by marbling scores) [9], residual feed intake [10] and iron content [11].

Using RNA-seq (mRNA sequencing) data, Cesar et al. [9] identified differentially expressed genes (DEGs) in muscle tissue and elucidated some of the molecular mechanisms involved in the lipid metabolism in Nelore steers breeding herd (castrated males, belonging a experimental population) genetically divergent for intramuscular fat (measured by marbling scores). When castrated and uncastrated Nelore males are compared, there are differences in intramuscular fat deposition [12, 13]. Therefore, gene expression and the molecular mechanisms regulating differences in intramuscular fat deposition may be different in castrated and uncastrated animals. Most commercially Nelore cattle produced in Brazil and exported to European courtiers are uncastrated. Therefore, understanding the biological and functional mechanisms that regulate IF content in uncastrated Nelore cattle is a compelling question in meat science, since this knowledge are still unclear.

The development of IF is influenced by different genes and a complex network of genetic interactions. The biological interpretation of the results obtained from the analysis of RNA-seq, especially for DEGs, remains a challenge. Additional studies are necessary, since many genes and mechanisms that induce differences in uncastrated Nelore male IF content are still unknown, especially hubs genes (biomarkers). Hub genes are highly correlated with a large number of genes, have been shown to play key regulatory roles in gene expression networks [14–16], and are proposed to play an important role in the overall biology of organisms [17]. Network approaches have been used to identify complex transcriptional regulation, i.e., in the identification of hub genes. Thus, co-expression analysis may facilitate the detection of important biological pathways involved in targeted phenotypes. Consequently, the aim of this study was to use transcriptome RNA-Seq data from *longissimus thoracis* muscle of uncastrated Nelore males to identify hub genes based on a gene co-expression network constructed from DEGs associated with IF content.

## Methods

### Sample collection and phenotype

All animals (*N* = 80), uncastrated Nelore males belonging to the same contemporary group (i.e., remaining together from birth until slaughter), were from the Capivara Farm, which participates in the Nelore Qualitas Breeding Program. They were reared on grazing systems (*Brachiaria* sp. and *Panicum* sp. forage and free access to mineral salt) and finished in confinement for approximately 90 days. The diet was based on whole-plant silage and mix of sorghum grain, soybean meal or sunflower seeds were used as concentrate, with a concentrate/roughage ratio from 50/50 to 70/30. The animals were slaughtered at a mean age of 24 months - all on the same day and under the same conditions.

*Longissimus thoracis* muscle samples were collected from an area between the 12th and 13th ribs of the left half of each carcass two times: 1) at slaughter, stored in 15-mL Falcon tubes containing 5 mL RNA holder (BioAgency, São Paulo, SP, Brazil) at − 80 °C until total RNA extraction was performed for RNA sequencing analysis; and 2) 24 h after slaughter for an analysis of the IF content.

IF content was quantified for all animals, through chemical method for total lipid content, according to the methodology described by Bligh & Dyer [18]. The animals were ranked in accordance with their IF phenotype and the samples derived from the animals with the 15 highest and 15 lowest values for IF content were selected for RNA-seq analysis (Table 1). A Student’s t-test was performed to evaluate whether there were significant differences in IF between the selected groups.

**Table 1:** Descriptive statistics for ribeye muscle area and intramuscular fat content of Nelore cattle

Phenotype*	N	Mean ± standard deviation	Minimum	Maximum	*p*-*value*
HIF	15	0.101 ± 0.0095	0.094	0.126	0.05
LIF	15	0.063 ± 0.0049	0.051	0.067	

*The data were transformed by the square root of the percentage of the arcsine function. HIF = Highest intramuscular fat content; LIF = Lowest intramuscular fat content; N = Number of animals

### RNA-seq library construction

Total RNA was isolated from the *longissimus thoracis* samples (an average of 50 mg each) using the RNeasy Lipid Tissue Mini Kit (Qiagen, Valencia, CA, USA) according to the manufacturer’s protocol. The following three methods were used for RNA quantification and qualification: RNA purity was determined by evaluating absorbance using a NanoDrop 1000 spectrophotometer (Thermo Fisher Scientific, Santa Clara, CA, USA; 2007); RNA concentration was measured using a Qubit® 2.0 Fluorometer (Invitrogen, Carlsbad, CA, USA; 2010), and RNA integrity was assessed using an RNA Nano 6000 Assay Kit with the Agilent 2100 Bioanalyzer (Agilent Technologies, Santa Clara, CA, USA; 2009).

Sequencing libraries were prepared using the TruSeq RNA Sample Preparation Kit® (Illumina, San Diego, CA) following the manufacturer’s protocol. Libraries were pooled to enable multiplexed sequencing and generated an average of approximately 25 M reads per sample. RNA sequencing (RNA-Seq) was carried out on a HiSeq 2500 System (Illumina®) that generated 100 bp paired-end reads.

### Data filtering and alignment of reads

Trimmed data (trimmed reads) were obtained by removing low quality reads (adapter sequence and reads containing poly-N) from raw data using Trimmomatic *v.*0.36 [19]. All downstream analysis was based on the trimmed data with high quality reads. HISAT2 *v.*2.0.5 [20] was used to align the paired-end trimmed reads to the bovine reference genome (UMD3.1.1 *Bos taurus*) and chromosome Y (Btau 4.6.1), both deposited in National Center for Biotechnology Information (NCBI) (https://www.ncbi.nlm.nih.gov/).

### Differentially expressed genes (DEGs) and enrichment analysis

The Cufflinks2 v.2.1.1 suite of tools [21] was used for transcriptome assembly and differential expression analysis. Cufflinks2 assembles transcriptomes from RNA-Seq data and quantifies their expression in fragments per kilobase of transcript per million reads mapped (FPKM). After assembly, the Cufflinks2 output per sample was merged together by Cuffmerge2. A uniform set of transcripts was obtained for all samples and then Cuffdiff2 was used to test for genes that were differentially expressed between the IF groups. The Cuffdiff2 uses the t-test to calculate the *p-values* for differentially expressed genes, based on the normalized FPKM values (log2FPKM) between two conditions (HIF and LIF groups). The method assumes that the data present normal distribution [22]. False discovery rates (FDR) were controlled using the Benjamini-Hochberg procedure in which we considered a gene to be differentially expressed if it had a *q-value* ≤ 0.05 [23].

The enrichment and pathway analyses of DEG sets was performed using the Database for Annotation, Visualization, and Integrated Discovery (DAVID 6.8) [24]. The DAVID Pathway was used to map the enriched pathways from the Kyoto Encyclopedia of Genes and Genomes (KEGG) database [25]. FDR were controlled by the Benjamini-Hochberg method [23] considering a *p-value* of less than 10% (EASE score ≤ 0.1).

### Co-expression network analysis and prediction of hub genes

All analyses were performed with plugins or applications from Cytoscape v.3.7, a free software package for visualizing, modeling, and analyzing the integration of biomolecular interaction networks with high-throughput expression data and other molecular states [26]. The co-expression network was constructed from the ExpressionCorrelation plugin [27]. The similarity matrix of count data from DEGs (normalized by FPKM) was computed using a Pearson correlation. A histogram tool was used for the screening criteria at node score cut-offs > 0.75 and < − 0.75 and employed to identify statistical significance of the pairwise correlations. We established a co-expression network between the significantly co-expressed DEGs.

To identify network modules, co-expression network was analyzed using Reactome FI plugin (cluster FI Network function) [28]. The modules within the network were mapped onto pathways and biological processes using the same plugin with analyze module function [28]. FDR were controlled by the Benjamini-Hochberg method [23] considering a *p-value* of less than 10%.

The cytoHubba plugin [29] was used to explore co-expression network nodes (hub genes). This plugin provides a user-friendly interface to explore important nodes in biological networks and computes using eleven methods (degree, edge percolated component, maximum neighborhood component, density of maximum neighborhood component, maximal clique centrality -MCC and six centralities - bottleneck, EcCentricity, closeness, radiality, betweenness, and stress). All these parameters can indicate the robustness of the analysis [29, 30]. MCC method has a better performance on the precision of predicting essential genes from the co-expression network [29] and to generate a subnetwork [30]. The top three hub genes were chosen to define top modules screened from the gene co-expression network. The top modules could be functionally relevant and therefore is used to assess biological function [30]. The Reactome FI Plugin [28] was then used to determine top modules- distinct groups of genes interactions [31] and to analyze the functional enrichment of biological pathways within the of each top module.

## Results and discussion

### RNA-seq data alignment

After quality control of the raw reads (approximately 25 million reads), the mean number of reads per sample (paired-end) was approximately 21.9 million. In the library, 88% of the clean reads were uniquely mapped to bovine reference genome UMD3.1.1 *Bos taurus* and chrY of Btau 4.6.1. The mean number of reads mapped in pairs using Hisat2 software was approximately 20.7 million (96.44%) with 45× sequencing coverage (coverage for all transcripts of all samples). The box plot containing the transformed FPKM values (log10) for each group and the plot of principal component analysis (PCA) were showed in Additional file 2: Figure S1. These plots were constructed using the cummerRbund package [32]. The distribution of quartiles, on box plot, was consistent between groups, indicating high quality of the data. In addition, the medians were similar in the two groups and close to − 1, indicating that the level of sequencing coverage permitted the identification of low-expressed genes [10]. PCA showed the formation of different groups (highest and lowest IF), indicating differences in the expression of genes between the HIF and LIF groups.

### Differentially expressed genes (DEGs) and enrichment analysis

A total of 65 DEGs (*q* ≤ 0.05) were found, including 21 upregulated genes and 44 downregulated genes (Table 2) in the HIF group. The genes were distributed across almost all bovine chromosomes in different proportions, except chromosomes 11, 14, 24, mitochondrial (MT), and Y. Important genes that play a role in lipid metabolism, muscle development and immune systems were differentially expressed. DEGs associated with lipid metabolism included: CDP-diacylglycerol synthase 1 (*CDS1*) and solute carrier superfamily genes (*SLC22A4* or *OCTN1* and *SLC2A3* or *GLUT3*), all of which were upregulated in HIF animals. Also were found regulatory genes: mediator complex subunit 25 (*MED25*) and forkhead box O1 (*FOXO1*), both downregulated in HIF animals.

**Table 2:** Differentially expressed genes in the *longissimus thoracis* muscle of Nelore cattle divergent for intramuscular fat content phenotypes

Gene	Locus	HIF (μ)	LIF (μ)	Fold change (log2)*	*q value*
*CDS1*	6:101195739–101,273,209	0.56	1.92	1.79	0.02
*TNC*	8:105965489–106,067,896	0.71	1.83	1.37	0.01
*BARX2*	29:33240583–33,256,128	1.49	3.60	1.28	0.04
*EGR1*	7:51438971–51,441,815	12.59	30.35	1.27	0.01
*CCL2*	19:16232955–16,234,822	9.64	21.62	1.16	0.01
*MXRA5*	X:138875004–138,904,034	1.06	2.20	1.06	0.01
*PCDH12*	7:54685624–54,703,271	1.10	2.18	0.99	0.01
*SLC22A4*	7:23382631–23,425,462	3.93	7.81	0.99	0.04
*SLC2A3*	5:101896620–101,909,565	4.74	9.39	0.99	0.01
*APLNR*	15:81734844–81,738,412	1.37	2.68	0.96	0.01
*FSCN1*	25:39292718–39,302,192	4.08	7.02	0.78	0.01
*COL18A1*	1:146989202–147,041,015	2.88	4.90	0.77	0.01
*CLDN5*	17:74749249–74,750,524	14.81	24.51	0.73	0.01
*CD93*	13:42244261–42,247,908	6.31	10.10	0.68	0.01
*MCAM*	15:30414471–30,422,213	5.15	8.12	0.66	0.04
*DLL4*	10:36577284–36,586,360	2.68	4.21	0.65	0.04
*BOLA*	23:28502516–28,506,343	41.94	65.42	0.64	0.01
*IER2*	7:13543658–13,548,059	4.94	7.62	0.63	0.05
*IER3*	23:28088454–28,089,726	10.11	15.49	0.62	0.04
*PLXND1*	22:56776197–56,825,067	2.38	3.58	0.59	0.01
*PPP1R3B*	27:24314781–24,323,720	13.34	19.54	0.55	0.04
*NFKBIA*	21:46065545–46,068,942	32.35	21.85	−0.57	0.04
*CRISPLD2*	18:10985131–11,050,908	13.29	8.90	−0.58	0.04
*ANKRD52*	5:57399491–57,415,909	9.99	6.62	−0.59	0.01
*MED25*	18:56632972–56,645,624	13.37	8.79	−0.61	0.04
*NT5C2*	26:23983197–24,080,607	33.01	21.57	−0.61	0.04
*FOXO1*	12:21915289–22,009,079	11.38	7.37	−0.63	0.01
*PMP22*	19:33357138–33,386,333	36.62	23.67	−0.63	0.01
*RPS6KA3*	X:130145854–130,305,125	90.58	58.17	−0.64	0.01
*GADD45A*	3:77972151–77,975,240	44.72	28.68	−0.64	0.01
*SFMBT1*	22:48444152–48,549,526	6.77	4.33	−0.65	0.04
*LOC786565*	7:54392624–54,437,291	39.05	24.83	−0.65	0.01
*CIPC*	10:89365865–89,381,544	55.74	35.31	−0.66	0.02
*LOC782855*	6:81188550–81,188,972	114.47	72.10	−0.67	0.02
*LOC104973907*	13:78185912–78,193,935	6.04	3.78	−0.68	0.01
*DNAJA1*	8:76107881–76,117,637	49.92	31.06	−0.68	0.01
*NFIL3*	8:87878330–87,893,188	21.56	13.36	−0.69	0.01
*TMCO3*	12:90698789–90,720,756	44.09	27.02	−0.71	0.01
*EEF1A1*	9:13230571–13,237,071	210.35	128.79	−0.71	0.04
*RGCC*	12:11625777–11,641,054	27.50	16.64	−0.72	0.01
*MAOA*	X:105380190–105,462,564	9.07	5.47	−0.73	0.02
*ARID5B*	28:18003670–18,195,880	12.92	7.75	−0.74	0.01
*SDC4*	13:74391486–74,412,899	35.88	21.36	−0.75	0.01
*CISH*	22:50320204–50,325,618	11.74	6.97	−0.75	0.01
*PDE4D*	20:18748597–20,326,423	52.28	30.49	−0.78	0.01
*ADAMTS20*	5:37102284–37,333,841	4.75	2.75	−0.79	0.01
*HSPH1*	12:29820753–29,844,227	12.76	7.39	−0.79	0.01
*KIAA1671*	17:67368397–67,541,098	8.79	5.08	−0.79	0.01
*CD160*	3:21711758–21,725,178	3.40	1.88	−0.85	0.01
*SRXN1*	13:60944182–60,951,049	9.83	5.29	−0.89	0.01
*LOC513548*	9:88273884–88,299,107	9.26	4.94	−0.91	0.01
*KLHL30*	3:118105157–118,112,080	17.70	9.10	−0.96	0.01
*TNFRSF12A*	25:2435179–2,441,777	64.37	32.72	−0.98	0.01
*CDKN1A*	23:10551754–10,568,782	27.80	13.85	−1.01	0.01
*MYBPH*	16:760047–769,469	79.15	39.33	−1.01	0.02
*SMPDL3A*	9:28811575–28,829,543	11.21	5.53	−1.02	0.01
*LOC104971692*	3:66997750–67,054,450	169.28	80.23	−1.08	0.01
*GFPT2*	7:772036–817,756	5.13	2.42	−1.08	0.01
*GNPTAB*	5:65912911–65,996,254	13.49	6.24	−1.11	0.01
*SPOCK2*	28:28304693–28,329,944	6.10	2.82	−1.11	0.01
*MICAL2*	15:40914779–41,195,753	21.93	9.98	−1.14	0.01
*RNF115*	3:21626658–21,706,296	86.33	39.05	−1.14	0.01
*LOC781186*	20:3132654–3,195,037	34.42	15.42	−1.16	0.02
*IL1RAP*	1:77187089–77,343,695	2.36	1.04	−1.18	0.01
*RGS2*	16:13041891–13,045,246	14.64	6.41	−1.19	0.01

*The fold-change estimates (relative expression) refer to the HIF group

*CDS1*, plays an important role in the synthesis of triglycerides. Qi et al. [33] elucidated the role of *CDS*, a key enzyme in phospholipid metabolism, in cellular lipid storage as well as in the differentiation of adipocytes. They demonstrated that *CDS1* may regulate the expansion of lipid droplets essential for adipogenesis. The *CDS1* up-regulation in HIF animals could contribute in the adipocytes differentiation of these animals.

*SLC22A4* (*OCTN1*) is a plasma membrane carnitine transporter (an organic cation transporter) which is an important component of the carnitine system. Adequate carnitine levels are required for normal lipid metabolism and are important for energy metabolism [34, 35]. *SLC2A3* (*GLUT3*), was classically defined as a neuronal glucose transporter due to its high level of expression and initial characterization in nervous tissue [36]. However, *SLC2A3* is also highly expressed in other tissues with high energy needs, including muscle [37, 38]. The animals in the HIF group had higher expression levels of genes associated with lipid metabolism, indicating that these genes may contribute to adipogenesis and the maintenance of lipid metabolism in HIF animals.

A few notable examples of transcription factors that regulate lipid metabolism include FoxOs proteins and hepatic nuclear factors (HNFs) [39]. The *FOXO1* gene (belonging to the FoxOs proteins family), is one of the most important transcriptional effectors, promoting the expression of genes involved in gluconeogenesis [40, 41]. *FOXO1* plays a role in hepatic lipid and lipoprotein pathways, potentially considered the central link for the regulation and coordination of insulin action on carbohydrate and lipid metabolism [42, 43]. Previous studies showed that lipogenesis is modulated by *FOXO1* through sterol regulatory element binding protein 1c (SREBP-1c) [42, 43].

Functional annotation of DEGs revealed biological processes GO terms such as translation regulatory and transporter activities. The regulatory genes, such as *MED25,* we suggest that these genes could be involved in modulation of others gene expression and thus influencing in intramuscular fat deposition. *MED25* is a transcription factor that belongs to a variable functional complex that controls the constitutive expression of genes. Rana et al. [44] showed that *MED25* is a cofactor of HNF4α (nuclear factor), i.e., *MED25* plays a vital role in the modulation of the transcriptional activity of HNF4α. HNF4α regulates genes that are responsible for lipid and drug metabolism, such as cytochrome P-450. These researchers also showed that down regulation of *MED25* impairs a specific set of HNF4α target genes, suggesting a role for *MED25* in metabolism and lipid homeostasis.

Gene: Symbol of the differentially expressed gene; Locus: location of the gene in the *Bos taurus* genome; HIF (μ): mean normalized counts from highest intramuscular fat content; LIF: mean normalized counts from lowest intramuscular fat content; Fold change (log2): FPKM values obtained for HIF and LIF; *q-value*: *p-value* adjusted.

GO terms significantly enriched in the biological processes and pathways categories for all DEGs found this study are shown in Additional file 1: Table A1. The GO terms were closely associated with cell regulation and may have a putative association with lipid and muscle metabolism, for example: Cellular response to fibroblast growth factor stimulus (GO:0044344). Two genes were related to this GO term: C-C motif chemokine ligand 2 (*CCL2*) and delta-like canonical notch ligand 4 (*DLL4*), both of which were upregulated in HIF animals. *CCL2* was associated with intramuscular adipocyte differentiation in Japanese Black cattle [45] and *DLL4* was associated with beef tenderness induced by acute stress in Angus cattle [46].

GO terms and pathways related to the immune and hormonal systems were also overrepresented, for example: antigen processing and presentation of peptide antigen via MHC class I (GO:0002474), immune response (GO:0006955) and insulin resistance (bta04931). Bola family member (*BOLA*), upregulated in HIF animals, was found in most GO terms significantly enriched in the biological processes category and pathways associated with the immune system (see Additional file 1: Table A1). *BOLA* has been associated with marbling in Hanwoo (Korean Cattle) [47], tenderness [7], and reproductive performance [48] in Nelore cattle.

Other gene found in the most GO terms related immune and hormonal systems was NFKB inhibitor alpha (*NFKBIA*), downregulated in HIF animals. Immune cells such as macrophages depend of the recognition of lipid ligands by membrane proteins, surface/extracellular, and intracellular immune receptors. Involvement of the lipid receptors triggers an immune response. Oxidized lipids activate nuclear receptors, which play a role in lipid homoeostasis and also regulate, for example, the immune response directed by *NFKB.* Activation of macrophages promotes the production of cytokines and the induction of acute phase response, accompanied by systemic lipid changes [49].

*NFKBIA* downregulation may contribute to intracellular the increase in 3′–5′-cyclic adenosine monophosphate (cAMP) binding levels. cAMP is known to have a significant role in adipogenesis [50] (other details will be discussed afterward) and thus may have effects on IF content differences in Nelore cattle, if cAMP plays a role in lipid systems. The *NFKBIA* gene was found in two GO terms and six pathways. Insulin resistance (bta04931) was among the pathways found. Insulin is synthesized by beta cells of islets of Langerhans in the pancreas and is the most important hormone in the regulation of energy metabolism. This hormone is essential for carbohydrate intake, protein synthesis, and fat storage [51]. Insulin resistance causes some disorders of lipid metabolism: increased triglyceride and decreased HDL levels [52]. This gene was previously reported to be associated with gain in crossbred beef steers [53].

### Gene co-expression network analysis and prediction of hub genes

**Fig. 1:**
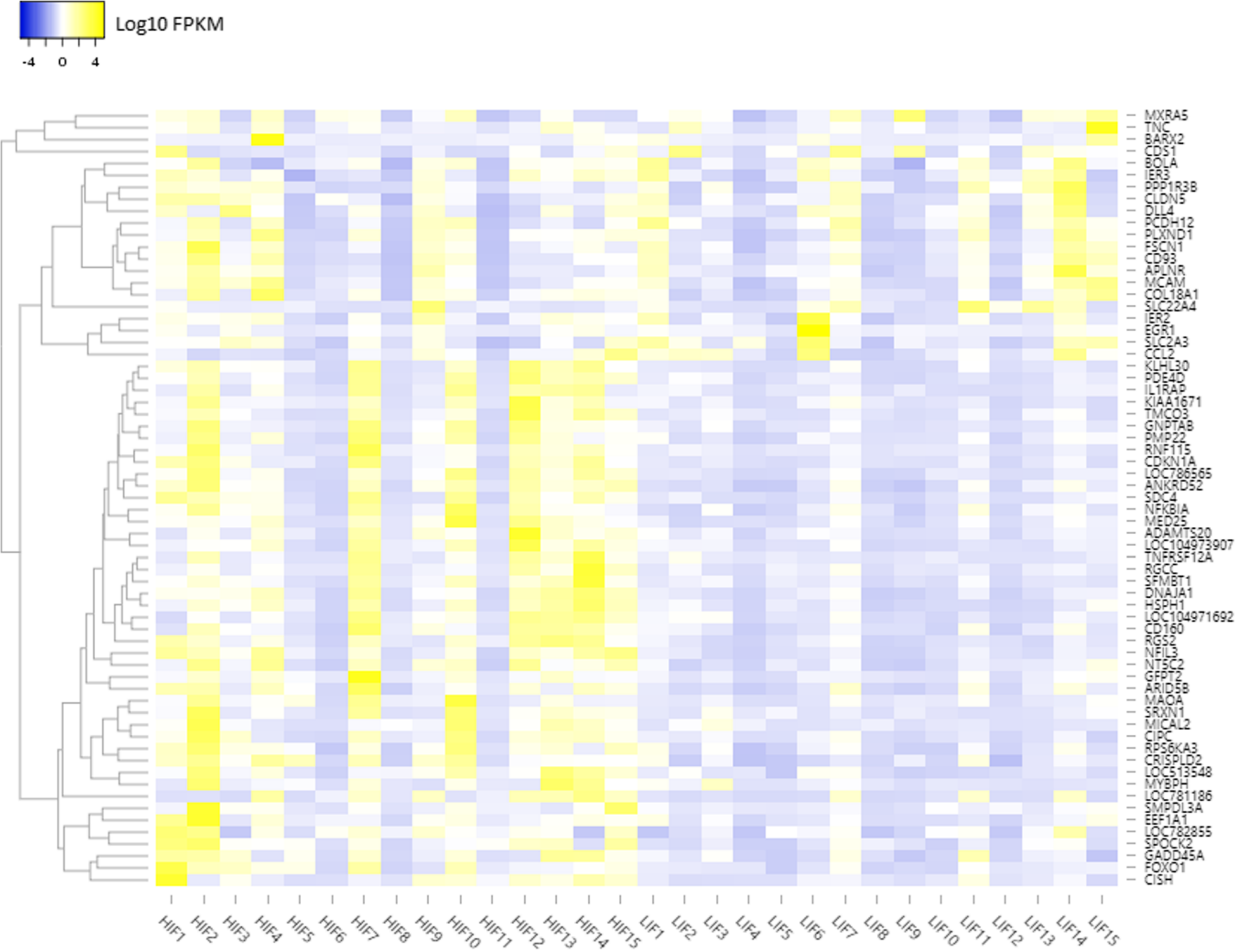
Heat map displays differentially expressed genes found in Nelore cattle divergent for intramuscular fat content. The differing colors represent differing levels of expression of those genes

**Fig. 2:**
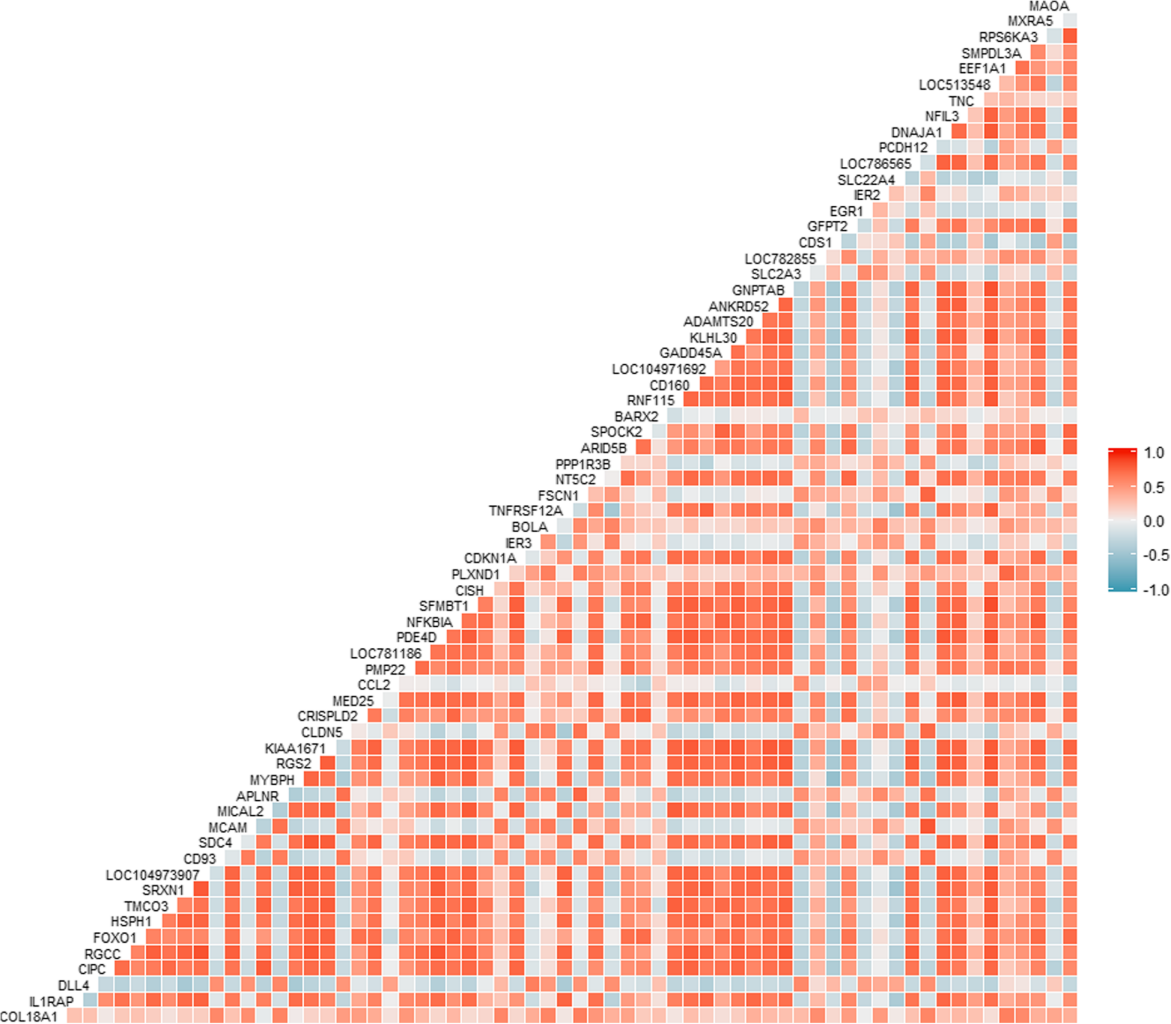
Correlation matrix plot. The differing colors represent differing levels of correlation between genes

**Fig. 3:**
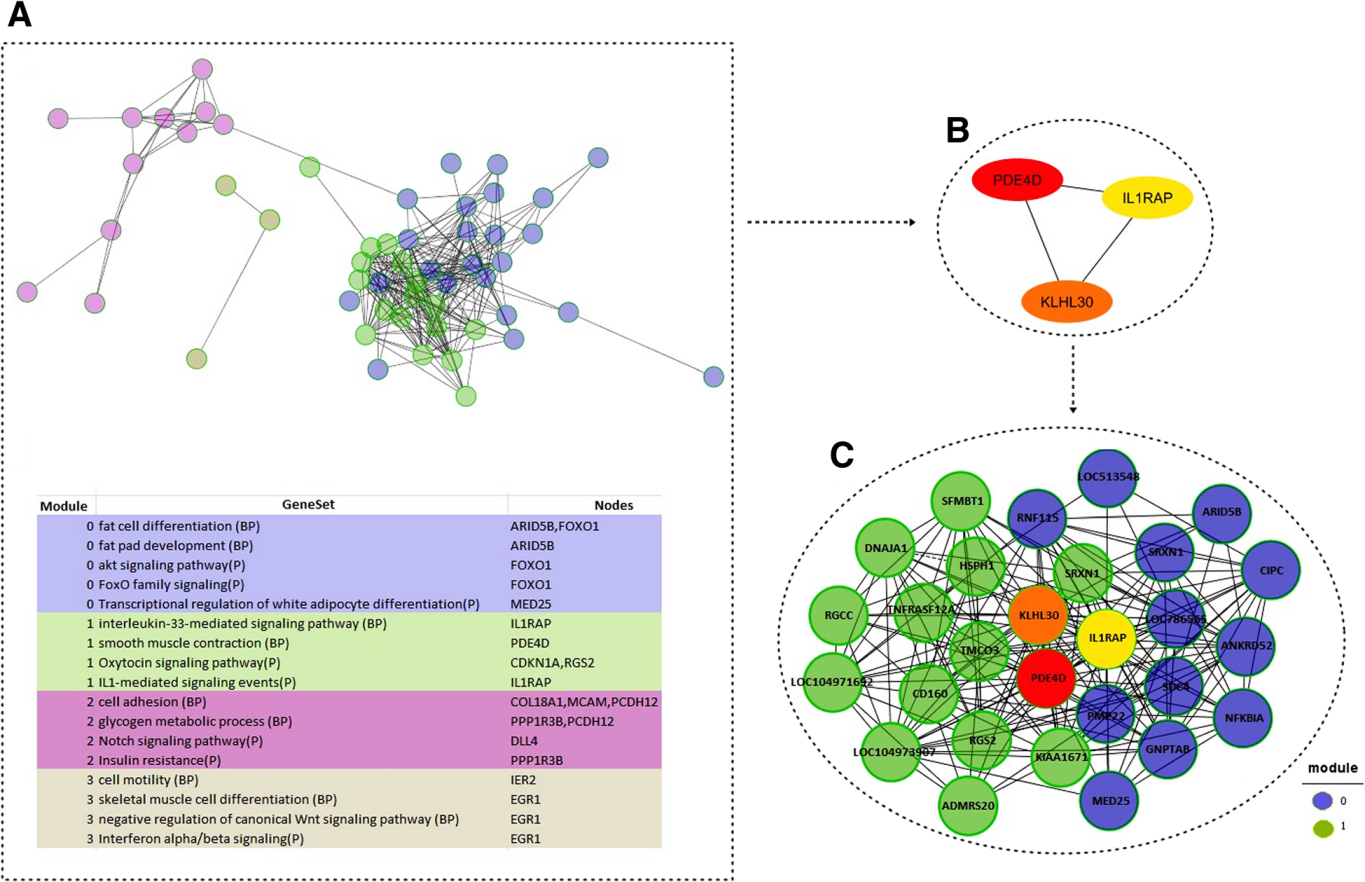
Gene co-expression network construction and module analysis. **a** Gene co-expression network. The nodes represent the differentially expressed genes involved in four modules (different colour code), the lines represent the interaction between nodes and summary of enrichment analysis of the modules (same colour code). **b** Top three hub genes with a maximal clique centrality -MCC. The more forward ranking is represented by a redder color. **c** Top modules screened from the gene co-expression network. The top three hub genes were chosen to define the subnetwork (top modules), as this represents the most functional elements of the network

The expression levels of transcripts changed dynamically between HIF and LIF animals as shown in the heat map (Fig. 1). We computed the correlation matrix using DEG expression profiles (Fig. 2). Most DEGs have a moderately high correlation, positive or negative. We used the correlation matrix to construct the co-expression network (Fig. 3). The network has 274 significantly correlated gene pairs (edges) that were discovered for 54 genes (nodes). From the co-expression network, module analysis of the components of the associated pathways could be carried out [31]. The parameters that indicate the robustness of the analysis [29, 30] are shown in Additional file 1: Table A2. The modules (same colour code in Fig. 3a; listed in Additional file 1: Table A3). were mapped onto biological processes and pathways. Four modules were identified within the Network, which had clear associated biological pathways. These include biological processes and pathways related to fat development, muscle contraction, cell adhesion, and muscle differentiation, among others (Fig. 3a, for more details see Additional file 1: Table A4).

Since we are looking for hub genes within the co-expression network, the first three genes in the MCC method, predicted by CytoHubba plugin (Fig. 3b), were chosen to define the subnetwork top modules (Fig. 3c, for more details see Additional file 1: Table A5). Two modules were identified within the subnetwork, which had clear associated biological pathways (for more details see Additional file 1: Table A6). The two candidate modules to contained considerable amount of genes and represent high intensity of the connectedness within the module in order to maintain high precision in their predictive capability. This analysis allowed the identification of genes that are related to lipid metabolism. The genes found this study, may therefore could to play a role as biomarkers of bovine IF.

Three top hub genes were predicted in this study were: phosphodiesterase 4D (*PDE4D*), kelch-Like family member 30 (*KLHL30*), and interleukin 1 receptor accessory protein (*IL1RAP*) (Fig. 3b). Hub genes, highly interconnected with nodes in a network, have been shown to be functionally significant [54], that is, the hub genes found may have a putative effect on lipid metabolism in Nelore cattle. According to our results, the *PDE4D* gene exhibited the highest centrality, indicating that it has influence on the other DEGs identified in this study, as such *NFKBIA*. *PDE4D* was downregulated in HIF animals and was present in module 1 of the subnetwork (Additional file 1: Table A5). This gene participated in more than 20 GO terms biological process and pathways in accordance to reactome FI analysis (FDR < 0.1), as for example: cAMP signaling pathway, regulation of cAMP metabolic process and positive regulation of interleukin-5 production (Additional file 1: Table A6), revealing that this gene also has effects on the immune system of the animals studied.

*PDE4D* belongs to a family of four *PDE4* genes, all encoding phosphodiesterase that hydrolyze the second messenger cAMP binding [55, 56]. The cAMP signaling pathways are among the well-characterized mechanisms controlling adipocyte differentiation [50] and immune system [57]. PDE inhibitors and synthetic cAMP analogs are commonly employed to switch on the adipogenic program in vitro [58]. *PDE4* genes can be regulated by transcriptional and/or by post-translational mechanisms [59–61]. This reflects a way of negative feedback for cAMP signaling, phosphorylation and activation of *PDE4* genes by the cAMP-dependent protein kinase (PKA) have been reported [59–61], that is, where *PDE4* is inhibited, cAMP levels are increased [57, 62]. This mechanism could putatively allow the rapid regulation of *PDE4D* activity in selected regions within cells and the impact on cellular functions requiring local *PDE4D* mediated control of cAMP levels [59]. Elevation of cellular cAMP concentration could, for example, influence early adipocyte differentiation [63].

As mentioned earlier, to maintain homeostasis, cyclic nucleotide levels are regulated by PDEs, with PDE4s predominantly responsible for degradation of cAMP, this regulation could have effects in inhibition activity of other promoters such as *NFKBIA*. *NFKBIA* was differentially expressed this study (downregulated in HIF animals) and was present in subnetwork module 0 (Fig. 3c).

Such effects on *NFKBIA* gene cause increased expression of anti-inflammatory signals and decreased mRNA expression of cytokines and other inflammatory mediators, such as interleukins. Therefore, cAMP signaling supports the maintain immune homeostasis by modulating the production of pro-inflammatory and anti-inflammatory mediators [57]. When intracellular cAMP concentrations are heightened, inflammatory signaling is reduced [57]. The fact that *PDE4D* was downregulated in HIF animals may increase the intracellular cAMP through the regulation of other genes and thus may contribute to an increase in IF content and maintain immune homeostasis of Nelore cattle. *PDE4D* was previously related with marbling score in a commercial Hanwoo cattle population from genome-wide association study (GWAS) [64].

*KLHL30* (downregulated in HIF animals) was identified as a hub gene and was present in module 1 of the subnetwork (Additional file 1: Table A5). This gene was not present in GO terms biological processes or pathways overrepresented this study. *KLHL30* belonging to the kelch (Klhl) superfamily that consists of a large number of structurally and functionally diverse proteins characterized by the presence of a kelch-repeat domain [65].

Klhl proteins are involved in a number of cellular and molecular processes such as cell migration, cytoskeletal arrangement, regulation of cell morphology, protein degradation (ubiquitination process), and gene expression [66]. The ubiquitin-proteasome system degrades the major proteins of contractile skeletal muscle and plays an important role in muscle loss [67]. Although *KLHL30* was not present in pathways and biological processes related to fat and the exact function that this gene has on production animal metabolism is still unknown, Piórkowska et al. [68] showed that *KLHL30* was differentially expressed in muscle tissue of chickens selected for differential shear force. This result, together with ours, shows the putative pleiotropic activities of *KLHL30* on qualitative traits in animals.

*IL1RAP*, downregulated in HIF, was identified as a hub gene encoding the interleukin 1 receptor accessory protein [69]. It is a necessary part of the interleukin 1 receptor complex which initiates signaling events that result in the activation of interleukin 1-responsive genes. Interleukins are a group of cytokines which play a role, primarily, in the immune system and in glucose/lipid metabolism [70]. Interleukins are expressed in a wide range of cells of the immune, neural and endocrine systems, reflecting the pleiotropic activities of this molecule [71]. The immune system can influence lipids and lipoprotein levels [72]. Lipids, besides being structural components of cellular membranes and serving as fuel stores, also play roles as effectors and second messengers associated with the immune system [73]. Interleukins can directly modulate lipid metabolism by increased of the activity of lipoprotein lipase, the enzyme that hydrolyzes triglycerides in lipoproteins. For example, the Interleukin-1 may inhibits adipocyte maturation and the synthesis of fatty acid transport proteins in adipose tissue in vitro [74].

*IL1RAP* was present in module 0 of the subnetwork (Additional file 1: Table A5). This gene participated in more than ten GO terms biological process and seven pathways in accordance to reactome FI analysis (FDR < 0.1), as for example: positive regulation of interleukins production, positive regulation of synapse assembly, protein complex assembly and inflammatory mediator regulation of TRP channels pathway (Additional file 1: Table A6). The elevation of cAMP can cause gene-specific inhibition of interleukin expressions and consequently of their receptors. The downregulation of *IL1RAP* in HIF animals may contribute to increase the intracellular cAMP and thus may to an increase in IF content and maintain immune homeostasis of Nelore cattle, as discussed before.

Of the hub genes identified from the coexpression analysis, only *IL1RAP* had colocalization with known QTLs controlling mainly reproductive traits of bovine (17 traits were associated, with 18 QTL/association found). This result, shows the putative pleiotropic activities of *IL1RAP* in producing qualitative traits in bovine.

We identify hub genes that play a role in lipid and muscle metabolism and immune system. These genes were the main regulators of others DEGs found this study and this promotes a better understanding of the essential biological mechanisms involved in IF deposition of Nelore cattle.

## Conclusions

Our results show that muscle cells of Nelore cattle phenotypically divergent for IF expressed genes related to lipid and muscle metabolism, as well as genes related to the immune system. Through gene co-expression network analysis, we identify modules and hub genes, which are the main regulators of other DEGs identified and consequently may play a role in Nelore cattle’s cellular or systemic lipid biology. We show that the fact that *PDE4D* was downregulated in HIF animals may increase the intracellular cAMP, and this, could influence adipocyte differentiation. The cAMP regulation could have effects in inhibition activity of other promoters such as *NFKBIA*.*,* and thus may contribute to an increase in IF content and maintain immune homeostasis of Nelore cattle. Such effects on *NFKBIA* gene cause increased expression of anti-inflammatory signals and decreased mRNA expression of cytokines and other inflammatory mediators, such as interleukins (*IL1RAP* was a hub gene and was downregulated in HIF, this gene was member of interleukins complex). Since, the interleukins can directly modulate lipid metabolism by increased of the activity of lipoprotein lipase, the downregulation of *IL1RAP* in HIF animals may decrease of the activity of lipoprotein lipase and thus may contribute to an increase in IF content in animals. This study identified potential biomarkers and molecular mechanisms involved in IF content difference in Nelore cattle.

## Additional files

Additional file 1: **Table A1.** Biological Process GO terms and pathways obtained with the DAVID software for differentially expressed genes in Nelore catlle muscle divergent for intramuscular fat content phenotypes. **Table A2.** Parameters from CytoHubba Plugin from Cytoscape software and hub genes. **Table A3.** Intramuscular fat associated modules as from co-expression network analysis. **Table A4.** Biological Process GO terms and pathways obtained with the Reactome FI Plugin from Cytoscape software for co-expressed genes network modules. **Table A5.** Intramuscular fat associated modules as from co-expression subnetwork analysis. (XLSX 75 kb)

Additional file 2: **Figure S1.** Global statistics and quality control. **a** Box plot of FPKM distributions for individual conditions. **b** PCA plot for gene-level features. (PDF 65 kb)

## Data Availability

The dataset utilized in this study belongs to a Qualitas Nelore breeding program company, and could be available on request. The author does not have authorization to share the data.

## Abbreviations

*BARX2*: BARX homeobox 2
*BOLA*: BolA family member
*CDS1*: CDP-diacylglycerol synthase 1
*CIPC*: CLOCK interacting pacemaker
*COL18A1*: Collagen type XVIII alpha 1 chain
DEGs: Differentially expressed genes
*EGR1*: Early growth response 1
FDR: False discovery rates
*FOXO1*: Forkhead box O1
FoxOs: Forkhead O box proteins
FPKM: Fragments per kilobase of transcript per million reads mapped
*GNPTAB*: GlcNAc-1-phosphotransferase subunits alpha/beta
GO: Gene ontology
GWAS: Genome-wide association study
HIF: Highest intramuscular fat content
*IER2*: Immediate early response 2
IF: Intramuscular fat
*IL1RAP*: Interleukin 1 receptor accessory protein
*KLHL30*: Kelch-Like family member 30
LIF: Lowest intramuscular fat content
MCC: Maximal clique centrality
*MED25*: Mediator complex subunit 25
*MYBPH*: Myosin binding protein H
NFIL3/ E4BP4: Nuclear factor and interleukin 3 regulated
*NFKBIA*: NFKB inhibitor alpha
*PDE4D*: Phosphodiesterase 4D
*SLC22A4* and *SLC2A3*: solute carrier superfamily genes
