# Supplementary material for: Prediction of hub genes associated with intramuscular fat content in Nelore cattle
Source: BMC Genomics. 2019 Jun 25;20:520. doi: 10.1186/s12864-019-5904-x (PMC6591902; doi:10.1186/s12864-019-5904-x)
Supplement: Supplementary file 2 — Figure S1. Global statistics and quality control. a Box plot of FPKM distributions for individual conditions. b PCA plot for gene-level features. (PDF 65 kb) [file 12864_2019_5904_MOESM2_ESM.pdf]

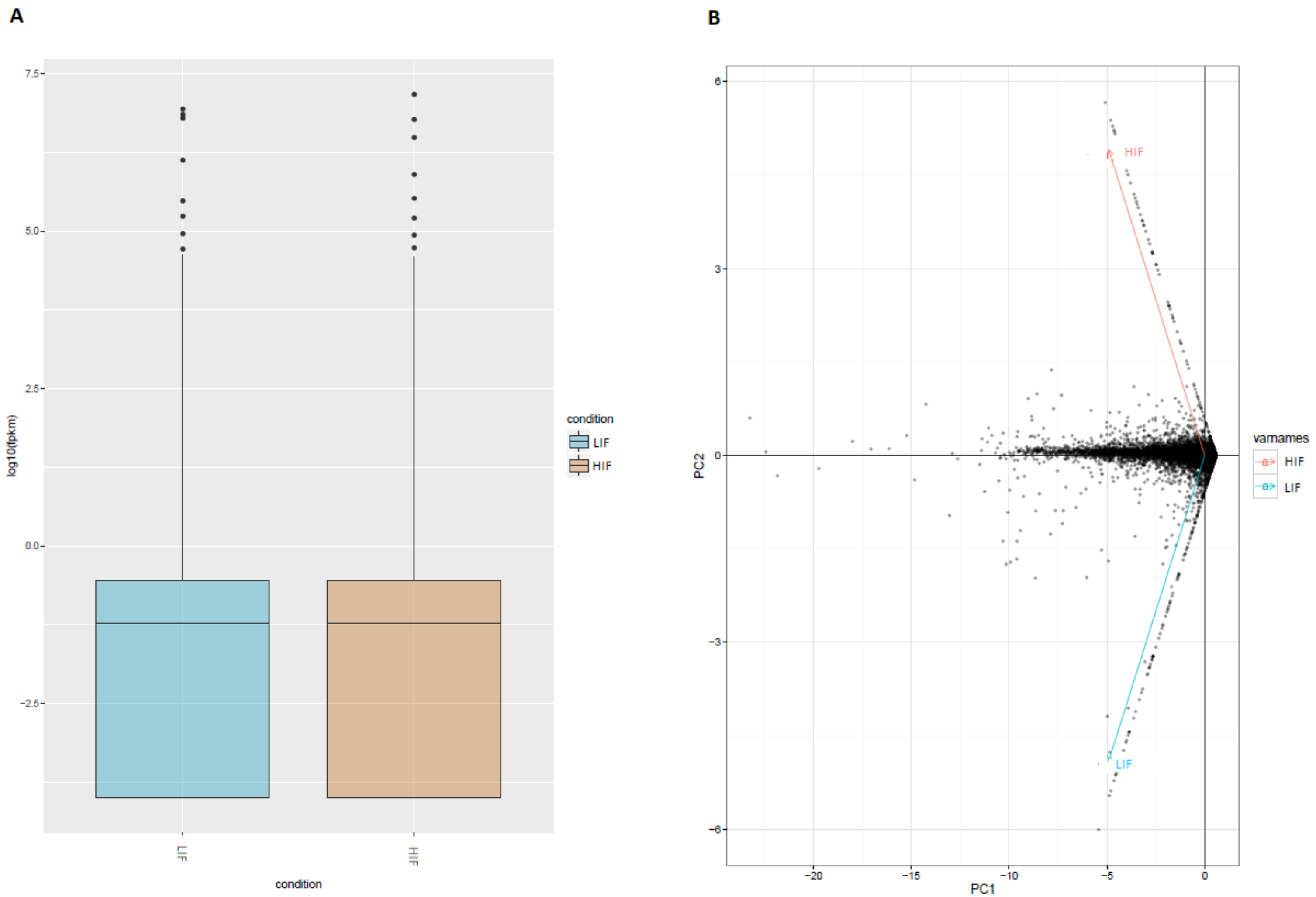

Additional Figure 1. Global statistics and quality control. (A) Box plot of FPKM distributions for individual conditions. (B) PCA plot for gene-level features.
